# Editorial: The impacts of iron accumulation on cognitive impairments

**DOI:** 10.3389/fnhum.2023.1198276

**Published:** 2023-04-27

**Authors:** Ana M. Daugherty, Bart Larsen, Nadja Schröder

**Affiliations:** ^1^Department of Psychology and Institute of Gerontology, Wayne State University, Detroit, MI, United States; ^2^Michigan Alzheimer's Disease Research Center, Ann Arbor, MI, United States; ^3^Department of Psychiatry, Penn/CHOP Lifespan Brain Institute, University of Pennsylvania, Philadelphia, PA, United States; ^4^Department of Physiology, Instituto de Ciências Básicas da Saúde, Universidade Federal do Rio Grande do Sul, Porto Alegre, RS, Brazil

**Keywords:** aging, dementia - Alzheimer's disease, ferroptosis, serum biomarker, imaging biomarkers

Iron is an essential micronutrient in the human diet that is absorbed and allocated throughout the body to support basic metabolic processes. Physiological iron can be classified by its function in the body: heme iron is a component of hem-containing proteins, such as hemoglobin for oxygen transport and cytochromes that are involved in mitochondrial energy production, and non-heme iron is stored in cells to support mitochondrial energy production and as cofactor for enzyme activity. In the nervous system, non-heme iron participates in myelination processes, neurotransmitter synthesis and receptor expression—the fundamental neural substrates of cognitive function. Whereas, large stores of non-heme iron are thought to support cognitive development in early life, iron dyshomeostasis and accumulation in aging and related disease coincides with incident neurodegeneration and functional loss. This set of observations has inspired a field of study to investigate the involvement of brain iron accumulation in the pathogenesis of neurodegenerative disorders and as a potential biomarker of risk for neural and cognitive declines.

Abnormal non-heme iron accumulation in brain tissue in aging and related disease has been suggested for over a century, with first observations beginning in the 1850–80's (Foley et al., 2022). Nearly a century later, the free radical theory of aging posited iron as an essential redox agent that is abundant in human tissue and precipitates oxidative stress, with potential downstream degenerative effects (Harman, 1956). Yet, up until recently, exploring the association of iron with neurodegeneration had been limited to post-mortem tissue assay. New pre-clinical models and clinical measurement has advanced experimental and *in vivo* observational studies that are necessary to validate use of iron as a biomarker of neural and cognitive decline. In particular, studies of cognition and function in relation to iron homeostasis across the life course are rare and this remains a critical gap for clinical translation. This themed issue includes summary review articles on iron dyshomeostasis potentially driving neurodegeneration, and empirical studies applying *in vivo* measurement to predict dementia and age-related cognitive decline.

Cumulative evidence indicates that iron accumulates in brain regions during typical aging and, to a greater extent, with the progression of neurodegenerative diseases, such as Alzheimer's and Parkinson's diseases. What remains unclear is if iron precipitates pathology or is a coincident biomarker of neurodegeneration. Wang et al. provide a scoping review article of iron dyshomeostasis that reciprocally interacts with multiple pathways of neurodegeneration, including glial activation and neuroinflammation, oxidative stress, and neuronal loss. Building from a strong pre-clinical evidence base, Wang et al. further link iron dyshomeostasis with amyloid β and hyperphosphorylated tau accumulation that are hallmark pathology of Alzheimer's disease. Ferroptosis—a unique form of cell death that is the result of iron-driven accumulation of lipid peroxides—may be a linchpin in Alzheimer's disease pathogenesis. They review areas for further study, including several promising routes of treatment to test a causal role of iron in Alzheimer's disease.

This future direction of study underscores the need for iron measurement techniques that can be correlated with changes in brain health and cognitive function, or that are sensitive to targeted intervention. Tran et al. review modern methods, including *ex vivo* assay, peripheral measurement, and *in vivo* imaging that can probe the intersection of iron dyshomeostasis and Alzheimer's disease pathogenesis. The multiple methods have been validated with correlations to disease pathology, cognitive decline or disease symptom progression, but each offer different perspective on the function of iron. Biofluid measurement of iron-related proteins and metabolites in cerebrospinal fluid, blood serum and saliva can estimate systemic iron dyshomeostasis, whereas *in vivo* magnetic resonance imaging provides brain region estimates of iron for specific cognitive vulnerability. They conclude there is insufficient evidence at this time to support iron dyshomeostasis as a cause of neurodegeneration in aging and related disease; however, its colocalization with neural pathology and correlation with functional impairment further bolster use of iron measurements as a biomarker for risk of cognitive decline.

The empirical papers in this themed issue further underscore this application. Ficiarà et al. apply machine learning profiles to demonstrate the value of biofluid iron as an additional biomarker integrated into the amyloid/tau/neurodegeneration framework of Alzheimer's disease diagnosis. As described in the included review articles, the accumulation and action of amyloid β, hyperphosphorylated tau and downstream neurodegeneration all act in concert with iron dyshomeostasis. Ficiarà et al. report that the addition of cerebrospinal fluid and serum iron measures improve identification of persons with dementia of the Alzheimer's type and mild cognitive impairment relative to control. This suggests biofluid markers of iron dyshomeostasis that are feasible in clinical settings may aid early detection and intervention for Alzheimer's disease risk. The sensitivity of iron measures to pre-clinical cognitive decline is further shown by Gustavsson et al. With *in vivo* quantitative susceptibility mapping, they find iron accumulation in the dorsolateral prefrontal cortex in healthy adults (ages 20–79 years at baseline) predicted greater decline in working memory over approximately 3 years. Intriguingly, the Val158Met polymorphism of the Cetechol-O-methyltransferase (*COMT*) gene modified the rate of iron accumulation among older adults in the sample: Val homozygotes, who presumably had lower endogenous dopamine, had greater iron accumulation in the dorsolateral prefrontal cortex and striatum as compared to Met carriers. Non-heme iron is involved in dopamine synthesis, and the novel work of Gustavsson et al. suggests that genetic predisposition for low endogenous dopamine may exacerbate iron accumulation in catecholaminergic regions that support age-sensitive working memory functions.

This themed issue provides a framework for future study into the application of iron as a biomarker of risk for neural and cognitive decline. Contained within is summary evidence from over 150 years of study, modernized methods, and avenues to probe the intersection of iron dyshomeostasis with other plausible mechanisms of cognitive impairment and neurodegeneration. Building from this set of papers, the field may one day answer the fundamental question: what is the role of iron in cognitive decline?

## Author contributions

AD, BL, and NS contributed to the writing and revision of this editorial, accompanying the curated, and edited themed issue. All authors contributed to the article and approved the submitted version.
